# Silver Nanoparticles in the Lung: Toxic Effects and Focal Accumulation of Silver in Remote Organs

**DOI:** 10.3390/nano7120441

**Published:** 2017-12-12

**Authors:** Martin Wiemann, Antje Vennemann, Franziska Blaske, Michael Sperling, Uwe Karst

**Affiliations:** 1 IBE R&D Institute for Lung Health gGmbH, Mendelstr. 11, 48149 Münster, Germany; vennemann@ibe-ms.de; 2 Institute of Inorganic and Analytical Chemistry, University of Münster, Corrensstr. 30, 48149 Münster, Germany; franziska.blaske@googlemail.com (F.B.); msperlin@uni-muenster.de (M.S.); uk@uni-muenster.de (U.K.)

**Keywords:** silver nanoparticle, quantitative bio-imaging, in vitro toxicity, nanoparticle transition

## Abstract

The distribution of silver (Ag) into remote organs secondary to the application of Ag nanoparticles (Ag-NP) to the lung is still incompletely understood and was investigated in the rat with imaging methods. Dose-finding experiments were carried out with 50 nm- or 200 nm-sized polyvinyl pyrrolidine (PVP)-coated Ag-NP using alveolar macrophages in vitro and female rats, which received Ag-NP via intratracheal instillation. In the main study, we administered 37.5–300 µg per rat lung of the more toxic Ag50-PVP and assessed the broncho-alveolar lavage fluid (BALF) for inflammatory cells, total protein and fibronectin after three and 21 days. In parallel, lung tissue was analysed for DNA double-strand breaks and altered cell proliferation. While 75–150 µg Ag50-PVP per rat lung caused a reversible inflammation, 300 µg led to DNA damage, accelerated cell proliferation and progressively increasing numbers of neutrophilic granulocytes. Ag accumulation was significant in homogenates of liver and other peripheral organs upon lung dose of ≥75 µg. Quantitative laser-ablation inductively-coupled plasma mass spectrometry (LA-ICP-MS) combined with enhanced dark field microscopy and autometallography revealed focal accumulations of Ag and/or Ag-NP in sections of peripheral organs: mediastinal lymph nodes contained Ag-NP especially in peripheral macrophages and Ag in argyrophilic fibres. In the kidney, Ag had accumulated within proximal tubuli, while renal filter structures contained no Ag. Discrete localizations were also observed in immune cells of liver and spleen. Overall, the study shows that concentrations of Ag-NP, which elicit a transient inflammation in the rat lung, lead to focal accumulations of Ag in peripheral organs, and this might pose a risk to particular cell populations in remote sites.

## 1. Introduction

The use of silver nanoparticles (Ag-NP) in consumer products is constantly increasing. Due to the high antibiotic activity of silver, the addition of Ag-NP is intended to counteract bacterial and fungal contamination of textiles, food packaging, cosmetics, implants or other medical devices [1,2,3]. The inflammatory, DNA-damaging and cytokine-inductive properties of Ag-NP have been well known for several years and have been reviewed along with innovative detection methods [4]. Consequently, many studies addressing safety aspects of Ag-NP have focused on the effects of Ag-NP secondary to oral, intravenous or intraperitoneal application [5,6,7]. However, as Ag-NP can also occur as airborne dust, e.g., during nanoparticle (NP) fabrication processes, well-adapted occupational exposure levels are necessary, as well, and have been recently suggested [2]. These considerations are based on inhalation and instillation studies conducted with Ag-NP of different sizes and surface coatings [8,9]. These studies show that Ag-NP in the lung exhibit a high inflammatory potential and can lead to granulomatous lesions. Moreover, it has become clear that Ag-NP applied to the lung lead to non-negligible concentrations of silver in remote tissues, most of all in liver, spleen and kidney, and especially in the liver, toxic effects were noted [10]. With respect to the biokinetic properties of Ag-NP, it appears reasonable to assume that small Ag-NP permeate the epithelial lung barrier and travel to remote organs via the blood and/or the lymphatic fluid [11]. However, due to the presence of oxygen, there appears to be also a contribution of dissolved silver ions, which may, at least in part, form secondary NP in the body [12,13,14,15]. Following the direct intravenous injection of Ag-NP, silver was qualitatively detected in phagocytic Kupffer cells of the liver (close to central veins or portal ducts) or in the kidney, where glomerulus capillaries, luminal brush border of proximal tubules or cortical perivascular regions were prominent deposition sites [16,17].

Recently, dissolved and nano-silver had been quantitated in organ lysates [18]. These studies suggested that after intravenous application, Ag-NP were mostly, but not exclusively, gathered in the liver [19], from where they were released into other organs where they were detectable as ions. Due to a disturbed liver metabolism, the authors suggested that silver NP toxicity was related to Ag-NP rather than to silver ions, a view substantiated by the finding that damage of endothelial cells and subsequent permeation through epithelial barriers is caused by Ag-NP rather than Ag ions [18]. However, as other studies pointed out that Ag ions rather than Ag-NP mediate cytotoxicity in vitro [20], ingested Ag-NP may become cytotoxic by releasing high local concentrations of Ag ions, which is commonly referred to as the Trojan Horse effect [21], which was recently verified via speciation analysis of silver inside cells [22]. Furthermore, a considerable contribution to the cytotoxic effects of Ag-NP seems to originate from oxidative stress generated by Ag-NP [23].

The aforementioned studies draw a complex picture of how Ag-NP and silver ions are distributed within the organism, making better and especially quantitative imaging methods highly desirable. Thus, although electron microscopy, enhanced dark field microscopy (DFM) [24] or autometallographic staining (AMG) can detect minute amounts of silver in single cells [17], they do not provide quantitative information of the silver concentration in tissue sections. Here, we used a quantitative approach to image silver in peripheral organs. To this aim, we employed laser-ablation inductively-coupled plasma mass spectrometry (LA-ICP-MS), which is a highly useful method to detect silver or other elements in cells or histological sections with high spatial resolution [25,26]. Importantly, LA-ICP-MS can be used to quantify Ag in tissue sections if appropriate standard materials are laser-ablated under identical conditions. With this approach, we quantified Ag in sections of peripheral organs of rats intratracheally instilled with Ag-NP and compared these results with images obtained from DFM and AMG, to distinguish between light scattering NP and AMG-positive silver species. To identify, for these studies, appropriate conditions of mild lung toxicity, the first part of the study is devoted to in vitro and in vivo dose-finding experiments and to the description of the respective toxic effects of Ag-NP in the lung.

## 2. Results

To find the appropriated dose window for instillation and subsequent elemental-imaging of silver NP in remote organs, we tested the cytotoxicity of polyvinylpyrrolidone (PVP)-coated Ag50-PVP and Ag200-PVP in vitro on alveolar macrophages (Section 2.1) and conducted a further dose-finding experiment based on in vitro results (Section 2.2). Silver distribution at the organ and cellular level is described for selected concentrations in Section 2.3.

### 2.1. Dose-Finding Studies with Silver Nanoparticles

#### In Vitro Experiments

**Figure 1:**
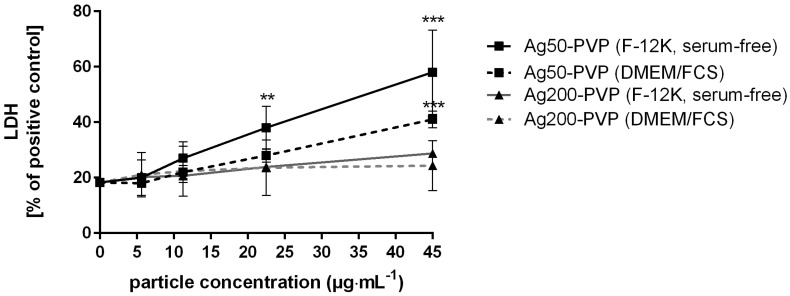
In vitro cytotoxicity of Ag50-polyvinylpyrrolidone (PVP) and Ag200-PVP. The activity of lactate dehydrogenase (LDH) released from rat alveolar macrophages (NR8383) into the medium is plotted against the concentration of Ag50-PVP and Ag200-PVP. Two different media and standard procedures were employed as described in the text. Note that cytotoxicity was increased upon 22.5 µg Ag50-PVP per mL, if particles were applied in serum-free F-12K medium (** *p* ≤ 0.01, *** *p* ≤ 0.001).

Cytotoxicity of Ag50-PVP and Ag200-PVP particles was tested by measuring the release of lactate dehydrogenase (LDH) from alveolar macrophages (NR8383) using two NP dispersion protocols: In the first protocol, tailored to be used with NR8383 macrophages, we dispersed Ag-NP in serum-free F-12K medium. In the second protocol, which is applicable to many cell lines, as it is conducted in the presence of serum, we dispersed NP by stirring in fetal calf serum (FCS)-supplemented (10% *v*/*v*) Dulbecco’s Modified Eagle’s Medium (DMEM) for 24 h. Both protocols yielded a dose-dependent release of LDH upon Ag-NP, whereby the effects of Ag50-PVP were notably larger than those of Ag200-PVP (Figure 1). Furthermore, the effects of the serum-free approach were more pronounced, although levels of cell controls were not different for both NP vs. controls. Significant effects of the serum-free approach commenced upon 22.5–45 µg. This concentration step is roughly equivalent to a mean cell loading of 15–30 pg/macrophage, because as previously shown [27], both types of Ag-NP form dark silver grains at the bottom of the cell culture, which were nearly entirely ingested by NR8383 cells. Of note, the hydrodynamic diameters of both Ag50-PVP and Ag200-PVP were highly similar in the absence and presence of serum (Table 1), suggesting that gravitational settling and uptake of particles by cells is similar.

### 2.2. In Vivo Experiments

To estimate a reasonable upper dose for intratracheal instillation experiments, i.e., a dose not overriding the lung clearance capacity by compromising alveolar macrophages, we multiplied the mean cellular dose at the beginning of cytotoxicity of the more potent Ag50-PVP (15–30 pg per macrophage) with the mean number of alveolar macrophages per rat lung (2 × 10^7^) and selected the dose of 600 µg of either Ag50-PVP or Ag200-PVP per rat lung to identify silver in blood and peripheral organs. No clinical signs were observed in treated rats or controls after three and 21 days. However, Ag50-PVP or Ag200-PVP led to severe signs of inflammation in broncho-alveolar lavage fluid (BALF) after three days: Percentages of polymorphonuclear neutrophilic granulocytes (PMN) were increased to approximately 70% of total cells (Figure S1). BALF contained assemblies of atypical cells and also some cell debris, possibly stemming from ruptured alveolar macrophages and/or deteriorated lung epithelium. Although effects on BALF had partially recovered on Day 21, and this effect was more pronounced for Ag200-PVP, lung histology still showed deteriorated or condensed alveolar septa with multicellular assemblies bearing numerous dark silver deposits (Figure S2). Dark Ag grains mostly occurred within remnants of macrophage-like cells often devoid of 4′,6-diamidino-2-phenylindole (DAPI)-positive nuclei (Figure S2). Due to these severe signs of tissue disruption, we continued the study with Ag50-PVP and an upper dose limitation of 300 µg per lung.

### 2.3. Lung Toxicity of Ag50-PVP Nanoparticles

#### 2.3.1. Analyses of the Broncho-Alveolar Lavage Fluid

**Figure 2:**
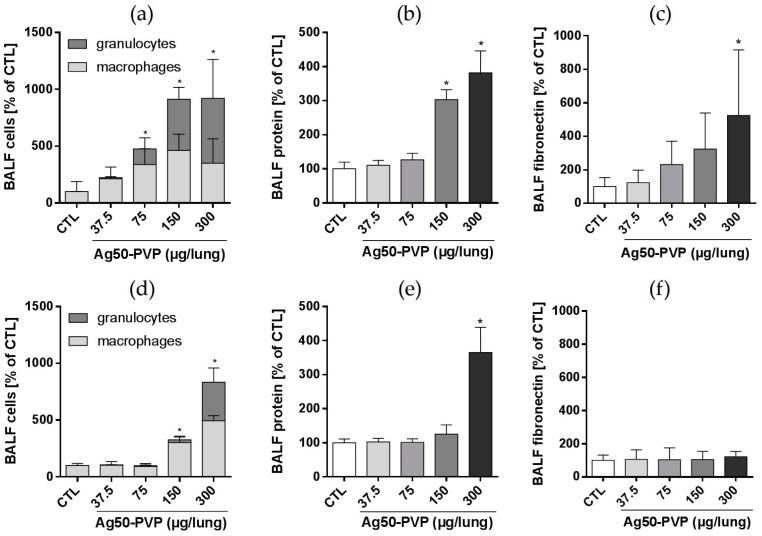
Effects of Ag50-PVP on broncho-alveolar lavage fluid (BALF) parameters of the rat lung. Indicated doses were intratracheally instilled, and effects were measured three days (**a**–**c**) and 21 days (**d**–**f**) post instillation in comparison to vehicle-treated controls (CTL). (**a**,**d**) Differential cell counts indicating increases in alveolar macrophages and neutrophilic granulocytes; (**b**,**e**) concentration of total protein; (**c**,**f**) concentration of fibronectin as quantified by enzyme-linked immunosorbent assay (ELISA). Values are the mean ± standard deviation (SD) from *n* = 5 rats; * *p* < 0.05 was revealed by one-way analysis of variance (ANOVA) followed by Dunnett’s multiple comparisons test.

Effects of intratracheal instillation of Ag50-PVP (37.5, 75, 150, 300 µg per rat lung) on BALF parameters are shown in Figure 2. With respect to increases in alveolar macrophage and PMN numbers, significant results vs. vehicle control were obtained upon 75 µg/rat lung. Total protein was increased upon 150 µg Ag50-PVP, and increases in fibronectin, which indicates extracellular matrix formation, became significant upon 300 µg/rat lung. While the effects of 75 µg/rat lung were fully reversed on Day 21 post application, 150 µg/rat lung further increased macrophage numbers, although PMN counts were reduced. In contrast, application of 300 µg Ag50-PVP progressively increased the numbers of alveolar macrophages and PMN, while protein concentration remained at a high level, indicating ongoing damage of the lung epithelium at this concentration.

#### 2.3.2. γH2AX Immunocytochemistry and Cell Proliferation

**Figure 3:**
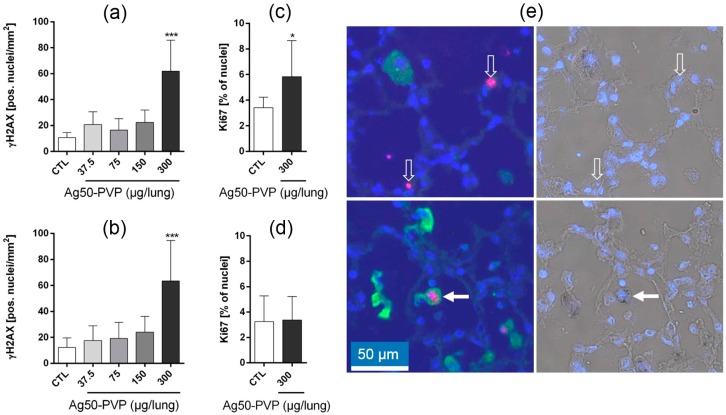
Effects of Ag50-PVP on double-strand breaks and proliferation in the rat lung. Phospho-histone H2AX (γH2AX) was detected by immunocytochemistry three days (**a**) and 21 days (**b**) post intratracheal instillation. The proliferation antigen Ki-67 was analysed three days (**c**) and 21 days (**d**) post application of 300 µg Ag50-PVP; (**e**) shows γH2AX-positive nuclei (red) in the lung parenchyma, together with CD68-positive alveolar macrophages (green) and DAPI-stained cell nuclei (blue). Left and right images in (**e**) show the fluorescence and bright field aspects of the same site. Note that γH2AX is expressed in nuclei within alveolar septa (open white arrows) and also in CD68-positive macrophages bearing numerous silver grains (white arrow). * *p* < 0.05, *** *p* < 0.001 as revealed by one-way analysis of variance (ANOVA) followed by Dunnett’s multiple comparisons test.

We furthermore analysed the lung parenchyma for double-strand breaks and proliferation using immunocytochemical detection of the phospho-histone γH2AX [28] and the proliferation marker Ki-67, respectively (Figure 3). The numbers of γH2AX-positive nuclei were unchanged up to 150 µg Ag50-PVP per rat lung. However, 300 µg Ag50-PVP led to an approximately six-fold increase of γH2AX-positive nuclei vs. vehicle control after three and 21 days. Double staining for γH2AX- and CD68-positive alveolar macrophage showed that epithelial cells and also alveolar macrophages, some of which contained dark silver grains, were immunopositive for γH2AX. At this concentration, cell proliferation, as detected by Ki-67, was transiently increased on Day 3, but no longer on Day 21 (Figure 3).

### 2.4. Peripheral Localization of Ag50-PVP

#### 2.4.1. General Organ Distribution of Silver

The general distribution of silver in blood and peripheral organs was quantified by conventional solution-based inductively-coupled mass spectrometry (ICP-MS) analysis after digestion of representative organ fragments. In the dose-finding experiment carried out with 600 µg Ag50-PVP per lung, blood values were transiently elevated to 30.0 and 21.1 ng/g on Days 3 and 21, respectively. In contrast, values (*n* = 3) increased in liver (Day 3: 7.2 ± 9.2 µg/g; Day 21: 16.4 ± 11.3 µg/g) and spleen (Day 3: 0.23 ± 0.2 µg/g; Day 21: 0.95 ± 0.76 µg/g). In kidney, 2.6 ± 1.9 µg/g were found on Day 21.

**Figure 4:**
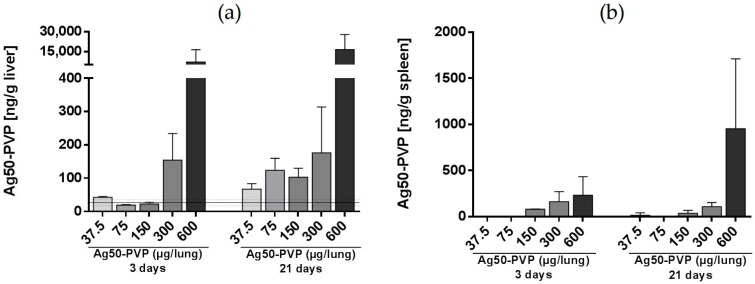
Time- and concentration-dependent changes of the Ag concentration in peripheral organs. Liver (**a**) and spleen (**b**) homogenates were analysed by inductively-coupled mass spectrometry (ICP-MS), three and 21 day after intratracheal instillation of 37.5–600 µg Ag50-PVP per lung. Data were pooled from two experiments; values are the means ± standard deviation from three animals per group. Solid and dotted lines show control values from non-treated animals.

The dose-dependency of silver deposition in liver and spleen (main experiment: 37.5–300 µg Ag50-PVP per rat lung) is shown in Figure 4a. After three days, increased amounts of Ag were found in the liver upon ≥300 µg Ag50-PVP per rat lung. After 21 days, all doses ≥37.5 µg Ag50-PVP per rat lung raised the Ag concentration in the liver, indicating a time-dependent accumulation of Ag at Ag-NP doses being non-toxic for the lung. In spleen, there was a dose-dependent increase of Ag starting at ≥150 µg per rat lung on Day 3. However, no aggravation of the Ag concentration over 21 days was noted in this organ, except for the 600-µg value (Figure 4b).

To further analyse the intra-organ distribution of Ag, organ sections of animals 21 days post intratracheal instillation of 150 and/or 300 µg Ag50-PVP were chosen for quantitative elemental imaging. From each group, sections from one or two animals being representative for the group (according to Figure 4) were analysed by LA-ICP-MS with a lateral resolution of approximately 50 µm (laser spot size).

#### 2.4.2. Laser Ablation Inductively-Coupled Mass Spectrometry of Liver Sections

**Figure 5:**
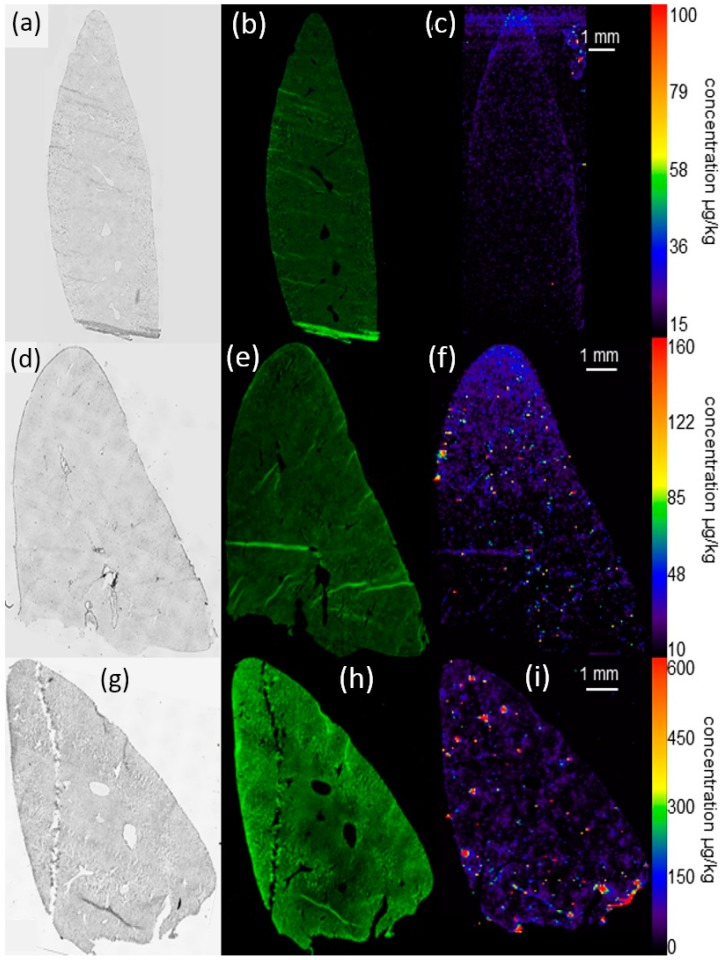
Distribution of silver in the liver 21 days post intratracheal instillation of Ag50-PVP into the rat lung. (**a**–**c**) Control animal; (**d**–**f**) 150 µg Ag50-PVP; (**g**–**i**) 300 µg Ag50-PVP. Low power micrograph (**a**,**d**,**g**), auto-fluorescence (**b**,**e**,**f**) and quantitative Ag distribution images determined by laser-ablation inductively-coupled mass spectrometry (**c**,**f**,**i**). Pseudocolor scales of the Ag concentration were adapted to organ concentration; maximum values of Ag are described in the text.

While the vehicle-treated control group showed a low and background signal in the liver parenchyma, which hardly exceeded 30 ng Ag/g, animals instilled with 150 µg of Ag-NP exhibited numerous focal increases not seen in controls (Figure 5). These silver accumulations mostly ranged from 80–160 ng/g with single peaks reaching up to 800 ng/g (not scaled in Figure 5). The diffuse background staining was qualitatively similar to controls, but slightly elevated (35–45 ng Ag/g). Rats instilled with 300 µg Ag50-PVP exhibited further intensified Ag accumulation with maximum values of up to 20 µg/g and an elevated background concentration. Thus, Ag50-PVP instilled into the lung led to focal accumulations of Ag in the liver, whose intensity rather than frequency reflected the augmented Ag burden of the organ.

#### 2.4.3. Laser-Ablation Inductively-Coupled Mass Spectrometry of Spleen Sections

**Figure 6:**
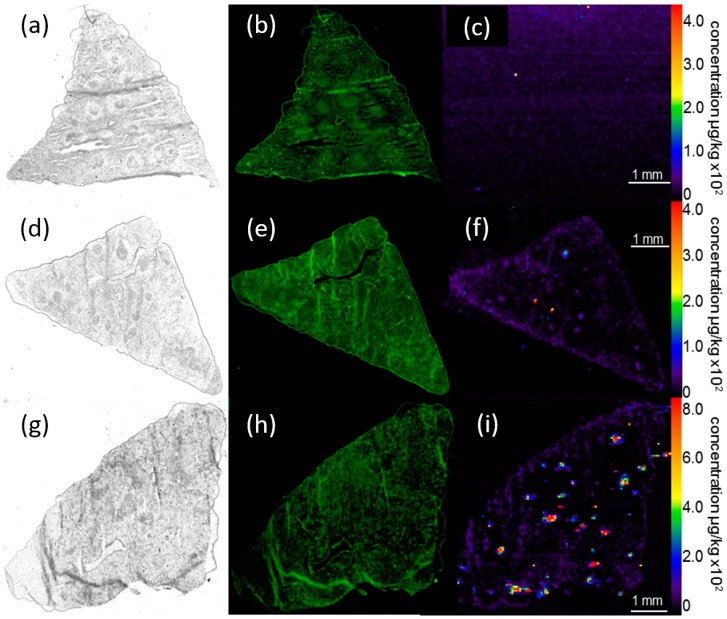
Distribution of silver in the spleen 21 days post intratracheal instillation of Ag50-PVP into the rat lung. (**a**–**c**) Control animal; (**d**–**f**) 150 µg Ag50-PVP; (**g**–**i**) 300 µg Ag50-PVP. Low power micrograph (**a**,**d**,**g**), auto-fluorescence (**b**,**e**,**f**) and quantitative Ag distribution images determined by laser-ablation inductively-coupled mass spectrometry (**c**,**f**,**i**). Pseudocolor scales of the Ag concentration were adapted to organ concentration; maximum values are described in the text.

While no Ag signal was detectable in transversal sections of the spleen from control animals, intratracheal instillation of 150 µg Ag50-PVP led to a low evenly-distributed Ag signal within the red pulp of air-dried (Figure S3) and formalin-fixed sections (Figure 6). In the latter, Ag concentrations ranged from 30–70 ng/g. Of note, many punctuate Ag accumulations of up to 1200 ng/g occurred within the white pulp and in the marginal zone (Figure 6). Treatment with 300 µg Ag50-PVP increased the mean silver concentration of the red pulp to 100–800 ng/g, while maximum concentrations and the number of local accumulations raised over-proportionally up to 34,000 ng/g.

#### 2.4.4. Laser-Ablation Inductively-Coupled Mass Spectrometry of Kidney Sections

**Figure 7:**
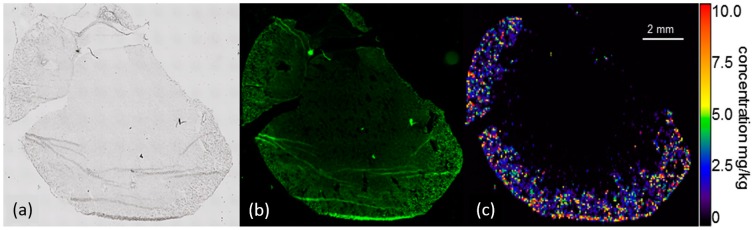
Distribution of silver in the kidney 21 days post intratracheal instillation of 300 µg Ag50-PVP into the rat lung. (**a**) Low power micrograph; (**b**) auto-fluorescence and (**c**) quantitative Ag distribution images determined by laser-ablation inductively-coupled mass spectrometry. Pseudocolor scales of the Ag concentration were adapted to organ concentration and do not show the maximum values mentioned in the text.

Longitudinal sections of the kidney from a rat 21 days post instillation with 300 µg of Ag-NPs revealed a pronounced Ag accumulation within the kidney cortex with very few Ag positive sites found also in sub-cortical regions of the medulla (Figure 7). Local Ag concentrations of the cortex ranged from 0.5–8 µg/g, and peak values reached up to 35 µg/g.

#### 2.4.5. Laser-Ablation Inductively-Coupled Mass Spectrometry of Mediastinal Lymph Node Sections

**Figure 8:**
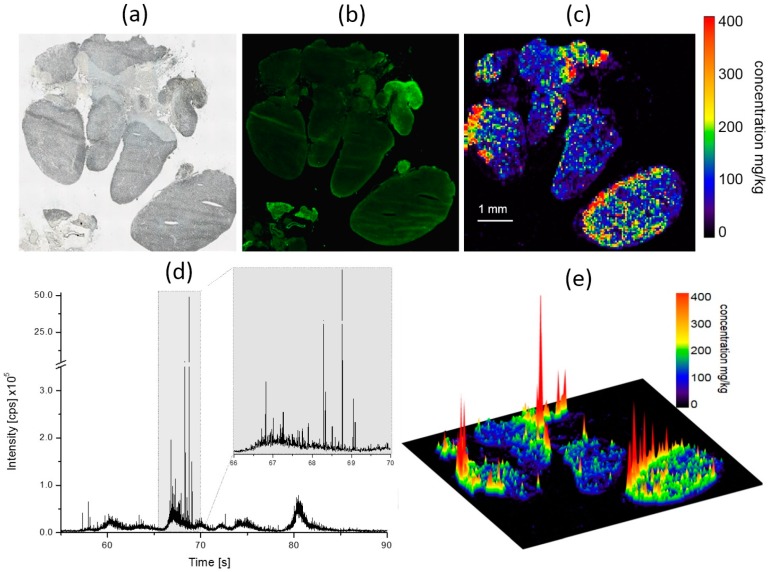
Distribution of silver in a collection of mediastinal lymph nodes 21 days post intratracheal instillation of 300 µg Ag50-PVP into the rat lung. Low power micrograph (**a**); auto-fluorescence (**b**) and quantitative Ag distribution images determined by means of laser-ablation inductively-coupled mass spectrometry shown as two- (**c**) and three-dimensional plots (**e**); (**d**) shows a (non-calibrated) single-line scan measured in counts per second (cps). High impact peaks represent aggregated or particulate Ag depositions. Pseudocolor scales of the Ag concentration in (**c**) were adapted to organ concentration. Maximum values are described in the text.

Mediastinal lymph nodes were collected from animals instilled with 300 µg of Ag50-PVP. As shown in Figure 8, Ag was found with a concentration of 50–150 µg/g in most parts of the lymph nodes, but not in surrounding fat tissue. In subcapsular and marginal areas, which are primary sites of invasion of lymphatic cells, Ag concentrations regularly amounted to 400 µg/g, as shown in Figure 8c,e. The maximum signal found in this tissue amounted to 4.7 mg/g (linearly-extrapolated value).

The high dynamic range of the Ag signal obtained by LA-ICP-MS in mediastinal lymph node sections is also shown as a non-calibrated line scan (Figure 8d). Of note, the ablation of Ag-NP as agglomerates or as single particles results in a high signal intensity compared to that of Ag ions. A first distinction between dissolved and particulate Ag was made by using the single-particle mode of the ICP-MS with a dwell time of 5 ms [29].

As shown in Figure 8d (boxed area), aggregated Ag gives rise to signal intensities of 5 × 10^6^ counts per second (cps), emanating from values of 0.3 to 0.5 × 10^6^ cps. In contrast, dissolved Ag appears related to a more consistent signal hardly exceeding 7.5 × 10^4^ cps.

#### 2.4.6. Detection of Ag-NP with Enhanced Dark Field Microscopy

**Figure 9:**
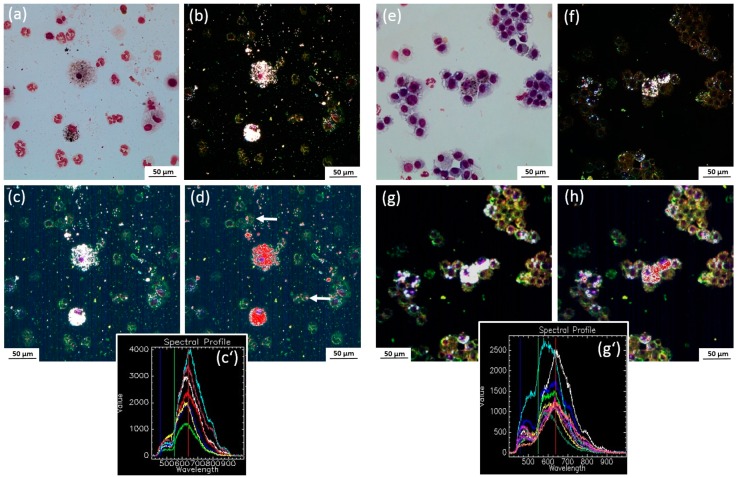
Identification of Ag50-PVP in Pappenheim-stained cytospin preparations from rat BALF. Rats were instilled with 150 µg Ag50-PVP per lung and lavaged after three (**a**–**d**) and 21 days (**e**–**h**). (**a**,**e**) Brightfield images showing alveolar macrophages and neutrophilic granulocytes; (**b**,**f**) corresponding dark field images; light-scattering material is contained in alveolar macrophages; (**c**,**g**) corresponding images from hyperspectral microscopy ; (**c’**,**g’**) spectral libraries of Ag50-PVP as collected from macrophages laden with light-scattering material; (**d**,**h**) matching of the spectral libraries as shown in **c’** and **g’** with the spectral angle mapping (SAM) method reveals Ag50-PVP (superimposed in red) in macrophages and, to a lower extent, in some neutrophilic granulocytes (arrows in **d**).

**Figure 10:**
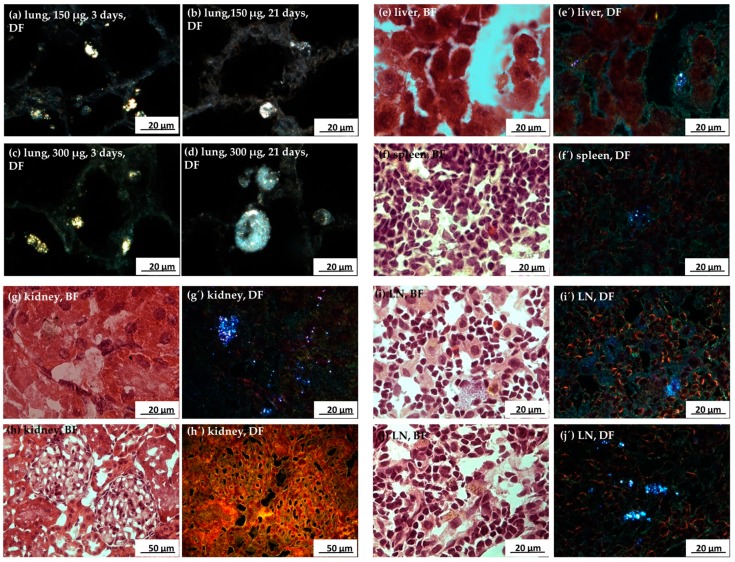
Enhanced dark field microscopy (DFM) of Ag50-PVP in rat lung and peripheral organs. All sections were from rats intratracheally instilled with Ag50-PVP. (**a**–**d**) DFM images (DF) from unstained lungs instilled with 150 µg (**a**,**b**) and 300 µg Ag50 PVP (**c**,**d**) after three days (**a**,**c**) and 21 days (**b**,**d**). (**e**–**j**) Hematoxylin eosin-stained sections of peripheral organs under bright field illumination (BF); (**e’**–**j’**) show the same regions under DFM illumination. In liver (**e**,**e’**), spleen (**f**,**f’**), and mediastinal lymph nodes (LN) (**i**,**i’**,**j**,**j’**), particles appear to be concentrated in large cells. In the kidney, light-scattering material is scattered or concentrated in the medulla (**g**,**g’**), but not in glomeruli or proximal tubuli (**h**,**h’**).

Previous studies of our laboratory have shown that the Ag50-PVP nanoparticles inside alveolar macrophages appear as prominent light-scattering objects when viewed with DFM. These particles can be further identified by hyperspectral microscopy (HSI). Typical DFM and HSI images from BALF of Ag50-PVP-laden lungs revealed numerous alveolar macrophages, but also PMN laden with Ag50-PVP (Figure 9). Similar Ag50-PVP-laden alveolar macrophages were found by DFM within lung parenchyma (Figure 10a–d). Of note, alveolar macrophages containing fine light-scattering material appeared swollen and/or enlarged on Day 21 (Figure 10d), suggesting an impairment of Ag-laden alveolar macrophages over time. In addition, numerous light-scattering particles occurred alongside and/or within alveolar septa (Figure 10a–d), and these structures were less frequent 21 days post instillation (Figure 10a,c).

In sections from liver, spleen, kidney and lymph nodes of Ag50-PVP-treated rats, DFM revealed bright, light-scattering nanoparticles within cellular structures not seen under control conditions (Figure 10e–j). Some of these cells exhibited dark inclusions and a brownish colour, when viewed in hematoxylin eosin (HE)-stained sections. Generally, the number of cells containing light scattering objects was low in all organs, and no major differences were noticed after three and 21 days. In the medulla of the kidney, cell-bound, as well as single scattered NP were occasionally found (Figure 10g). Interestingly, neither glomeruli, nor proximal tubuli of the kidney cortex contained these light-scattering structures (Figure 10h). Similarly, the marginal region of mediastinal lymph nodes was devoid of light-scattering material. Together, these results show that the high silver concentration found by LA-ICP-MS in these organs was not paralleled by the occurrence of light-scattering material in DFM.

#### 2.4.7. Autometallographic Detection of Silver

**Figure 11:**
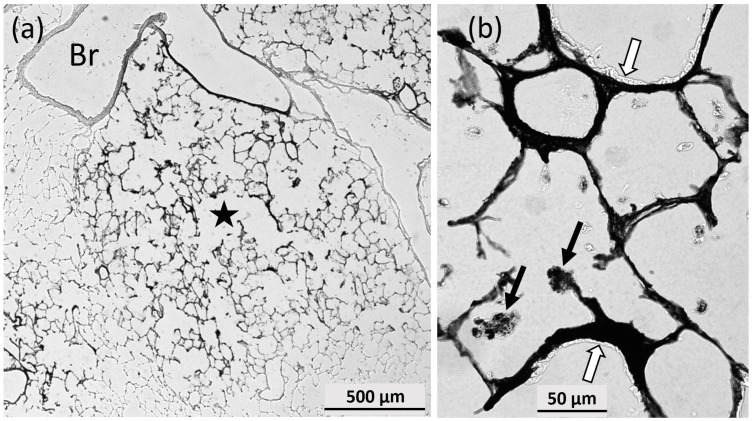
Autometallographic staining of a rat lung instilled with 150 µg Ag50-PVP nanoparticles. (**a**) Representative part of the lung epithelium; the asterisk marks a silver-containing region with black-stained alveolar septa; (**b**) at higher magnification, single alveolar macrophages (arrows) and densely-stained epithelium are visible. Open arrows point to unstained bronchial tissue.

**Figure 12:**
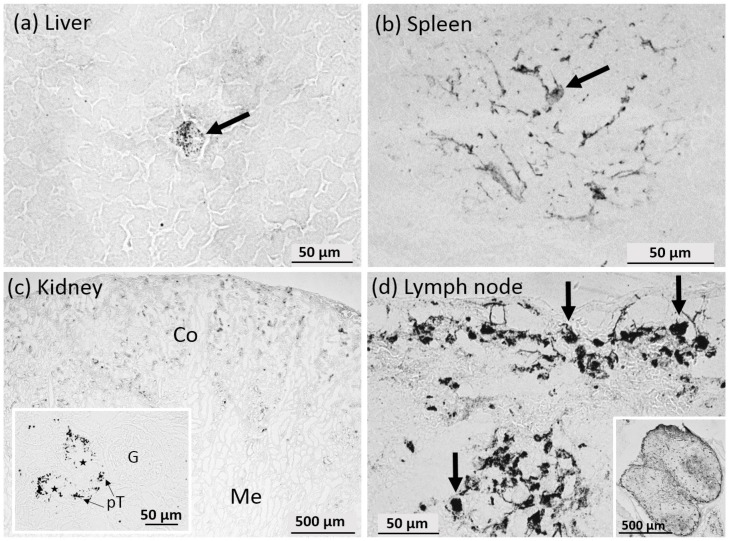
Autometallographic (AMG) staining of organ sections from a rat intratracheally instilled with 150 µg Ag50-PVP nanoparticles. (**a**) Liver parenchyma with dark silver grains concentrated in a cell (arrow) and some fine silver grains scattered throughout in the parenchyma; (**b**) spleen section with a typical roundish assembly of AMG-positive fibres centred by a cellular element (arrow); (**c**) kidney section showing representative parts of the cortical (Co) and medullary region (Me); (inset) the pronounced AMG-staining of the cortex is confined to proximal tubuli (pT, arrows), whereas glomeruli (G) are unstained.; (**d**) mediastinal lymph node; AMG-positive macrophage-like cells (arrows) occur in the marginal sinus and in the depth of the organ. The overview (inset) shows a prominent marginal staining and stained fibre elements spanning of two lymph nodes gathered for sectioning.

To further attribute the inhomogeneous silver distribution measured by LA-ICP-MS to cellular or tissue structures, we employed AMG staining of silver. This method converts the smallest amounts of Ag ion species into a prominent black stain and/or into silver grains. As such, AMG staining is not a quantitative method. Typical AMG staining results from the same animals analysed with LA-ICP-MS and DFM are shown in Figure 11 and Figure 12.

In the lung, intensely-stained alveolar macrophages were found in those regions of the lung parenchyma that exhibited a largely uniform positive AMG staining (Figure 11a,b). The overall distribution pattern of positive AMG staining was characteristic for the distribution of instilled particle suspension. Border zones between stained an unstained parenchyma showed a staining gradient. Interestingly, some elements of the parenchyma such as bronchial epithelium were excluded from AMG stain, even in the direct vicinity of AMG positive epithelium (Figure 11b), suggesting a cell-specific uptake of silver ions.

In the liver, there were focal assemblies of silver grains similar to macrophage-like single cells seen by DFM (Figure 12a). These cells were often surrounded by or adjacent to tissue with lower AMG staining intensity, reflected by less and smaller silver grains, which were hardly attributable to single cellular elements.

On spleen sections, prominent AMG positive sites appeared as roundish or oval-shaped areas with a diameter of several hundred microns. Higher magnification revealed a fine fibrous network, which encompassed cellular elements of still unclear identity (Figure 12b).

In the kidney, AMG staining led to distinct silver grains located nearly exclusively within the cortex alongside the brush border of the proximal tubuli and/or within the underlying epithelial cells (Figure 12c, inset). Bowman’s capsules or glomeruli remained unstained, demonstrating that Ag50-PVP NP did not accumulate at renal filter structures. Few AMG-stained grains also appeared in the medulla (Figure 12c).

In lymph nodes, AMG led to a complex staining pattern, which comprised heavily-laden phagocyte-like cells mainly located in the periphery and the marginal sinus and also in the depth of the organ. In addition, AMG positive fibre elements were stained throughout the organ (Figure 12d). The overall intensity distribution of the AMG stain with its prominent marginal staining (Figure 12d, inset) was in line with the Ag distribution quantified by LA-ICP-MS.

## 3. Discussion

In this study, we have shown that the application of silver nanoparticles (Ag50-PVP) to the rat lung in doses leading to transient lung inflammation and to the beginning of genotoxic effects is accompanied by the focal deposition of silver in lymph nodes, liver, spleen and kidney. To the best of our knowledge, this is the first study that has quantified focal depositions of silver by LA-ICP-MS in several organs and further compared results with both enhanced DFM and AMG staining.

### 3.1. Discrepancy between In Vitro and In Vivo Testing

Measuring LDH release from silver NP-treated alveolar macrophages indicated the macrophage toxicity of these NP, which strongly hints at the lung toxicity of the investigated NP [30]. However, the in vitro test carried out with a serum-free protocol was more sensitive than a test with the same cells subjected to Ag50-PVP suspended in the presence of serum and stirred for 24 h. This difference may be based on protein binding to the NP surface and/or on complexation of Ag ions by sulfhydryl groups of serum proteins. A mitigating influence of serum has been documented also for other nanoparticles in vitro, such as amorphous SiO_2_ [31]. Trying to extrapolate the lung burden leading to mild toxicity from the lowest toxic in vitro dose via the alveolar macrophage number per lung, a procedure which had yielded an acceptable approximation in instillation experiments, e.g., with ZrO_2_ NP [32], apparently overestimated the low observed adverse effect level (LOAEC). It appears possible that macrophages are less sensitive to Ag-NP or Ag ions than lung epithelial cells, but this assumption is contradicted by our own and other experiments in which phagocytic cells accumulating NP reacted more sensitively than non-phagocytic cells [33]. Other in vitro studies with epithelium forming T84 cells have shown that 20–100 µg/mL especially of small (10 nm) Ag-NP disrupt the epithelial barrier. As the effect was inhibited by EGTA (ethylene glycol-bis(2-aminoethylether)-*N*,*N*,*N*′,*N*′-tetraacetic acid), free Ag ions may be involved [34]. The pivotal role of Ag ions for cytotoxicity of Ag-NP is also suggested by the fact that acute cytotoxic effects of aged Ag-NP suspensions can be abrogated by dialyzing such preparations [20]. For instillation experiments, as performed here, it appears plausible that soluble Ag ions, together with even the smallest Ag-NP, are concentrated at the lung epithelium where the instilled fluid is resorbed, which leads to high local concentrations. In contrast, the access of Ag ions and very small Ag-NP to cells at the bottom of a culture vessel is largely diffusion limited. However, it is unlikely that the discrepancy of in vitro and in vivo findings is solely based on silver ions present in the instillation fluid, because particle-diminished supernatants, which equalled the highest possible dose of silver ions and which were applied as a control, had no effect on the lung (see Figure S1). Recent studies have shown that PVP-coated Ag-NP preferentially destroy endothelial cells when applied intravenously, a process involving the disruption of tight junction proteins [11]. In line with this, we found a pronounced damage of the lung epithelium at sites where engulfed Ag50-PVP or Ag200-PVP NP were deposited, and this form of damage persisted for at least 21 days (see Figure S2). It is also conceivable that the high oxygen pressure in lung tissue favours the release of Ag ions from Ag oxide. Considering all this information, we suggest that especially Ag ions are involved in tissue disruption and that these ions are released from Ag-NP gathered by alveolar macrophages. This process appears to damage alveolar macrophages over time, as suggested by the numerous swollen macrophages observed in the Ag50-PVP NP-laden lung parenchyma after three weeks. In vitro studies of slowly-dissolving Ag-NP should, therefore, consider prolonged exposure times to better reflect the toxicity of Ag-NP in tissue.

### 3.2. Toxic Effects of Ag50-PVP In Vivo and Selection of Appropriate Doses for Bio-Imaging Experiments

Concerning the toxic effects, our experiments revealed two concentration windows. The first one ranged between 75 and 150 µg per rat lung, where an initial, though reversible inflammation occurred, indicated by increased numbers of PMN and alveolar macrophages in BALF, accompanied by elevated protein concentrations. Compared to what was observed upon inhalation of Ag-NP, the low observed adverse effect level (LOAEL) of 75 µg found here appears comparatively high. Thus, the lung burden measured after a 90-day inhalation period was 23.54 µg per lung, as calculated from published values for the female rat study group [10]. This lower LOAEL concentration may be explained, however, by a partial dissolution of Ag-NP and the ongoing lung clearance.

The second dose window started around 300 µg Ag50-PVP per rat lung. Here, effects on BALF parameters turned out to be progressive or hardly reversible within the 21 day-long observation period. Notably, these effects were accompanied by an initial cell proliferation and, even more important, a persistent six-fold increment in DNA double-strand breaks, as indicated by γH2AX-positive nuclei. As γH2AX-positive nuclei were found in Ag50-PVP-laden alveolar macrophages and also epithelial cells without apparent Ag-NP load, direct and indirect genotoxic mechanisms may be involved. In line with these findings, several reports from the literature support the view that Ag-NP exert genotoxic effects in various cell types and that these effects are detectable with γH2AX immunocytochemistry [35,36,37,38], although some studies failed to demonstrate genotoxic effects of Ag-NP [21,39]. Nevertheless, the majority of studies that identified DNA damage speculated that this is due to Ag ions released from intracellular Ag-NP, an effect commonly referred to as the Trojan Horse effect [21]. Of note, positive γH2AX staining has received increasing interest as an early genotoxic marker with a high potential to predict cancerogenesis [40], and this has also been demonstrated for hazardous particles in the rat lung, such as quartz DQ12 [41].

Concerning the genotoxic dose of Ag-NP in vivo, two recent rodent studies should be mentioned for comparison, although Ag-NP were administered intravenously: The first one was carried out in mice where 5 mg Ag-NP per kg body weight (approximately 100–150 µg/animal) significantly increased DNA strand breaks in lung and other organs [42]. The second one was carried out in rats, also dosed with 5 mg Ag-NP per kg body weight (approximately 1000–1500 µg/animal), which led to organ levels of 10–15 µg Ag per g wet weight, as measured in lung, liver, spleen and kidney after 24 h [43]. Interestingly both, Ag-NP and the corresponding Ag^+^ ion fraction similarly increased the numbers of cells with aberrant chromosomes in bone marrow. In summary, the dose of 300 µg Ag50-PVP per rat lung, as used here, should be regarded as being genotoxic, at least in the lung.

### 3.3. Silver Distribution in the Body upon Instillation of Ag50-PVP into the Lung

#### 3.3.1. General Aspects

Previous studies have shown that Ag-NP applied intravenously (i.v.) or intraperitoneally (i.p.) are most likely accumulated in the liver, spleen and kidney [5,10,44,45,46]. In contrast, application of Ag-NP to the lung via inhalation, as well as instillation leads to massive loading of alveolar macrophages [45]. Nevertheless, Ag apparently leaves the lung and enters peripheral organs [2,10]. Using the data from a90-day whole body inhalation study [10] with aerosolized silver NP (2–65 nm), we calculated that from the 21.6 µg of Ag-NP (female rat group), only 0.35% and 0.18% of the total Ag lung burden was transferred into liver and kidney, respectively. While the lung developed typical signs of inflammation (perivascular mixed cell infiltration and macrophage hyper-cellularity), bile duct hyperplasia was a prominent abnormality in the liver [10]. Here, we analysed the organ distribution after the lung had been instilled with either 150 or 300 µg Ag50-PVP, i.e., under conditions of transient inflammation or inflammation plus genotoxic effects. Because the administration of 150 or 300 µg Ag50-PVP led to a transfer into liver and kidney of only 0.76% or 0.7% of the total Ag lung burden, respectively, we believe that the transfer of Ag into secondary organs reflects physiologically-relevant processes rather than artificial translocation caused, e.g., by resorbed instillation fluid. Of note, organs from the high concentration group (600 µg Ag50-PVP per lung) used for initial dose finding were not subjected to bio-imaging due to some sites with highly compromised lung epithelium (see Figure S2), whose leakiness might have caused the pronounced transfer of Ag, such that approximately 30% of the total lung burden was displaced into the liver and kidney.

#### 3.3.2. Organ Distribution of Silver

With respect to the elemental-imaging, the most striking finding of this study was that, under conditions of transient inflammation, Ag distributed not only to lung-draining lymph nodes, but also led to focal accumulations in liver, spleen and kidney, detectable by quantitative LA-ICP-MS. The laser spot size of 50 µm was a useful compromise for scanning whole organ sections, although it does not allow one to measure single cells, such that Ag-containing cells and/structures were analysed in parallel tissue sections by DFM and AMG. For the simultaneous interpretation of these results, it should be kept in mind that LA-ICP-MS quantifies Ag regardless of its ionic or particular form, while the method of enhanced DFM as used here detects single Ag-NP down to a particle size of approximately 20–30 nm [47], and AMG amplifies silver ions bound, e.g., to sulphide and selenide [48]. Thus, given the size distribution of Ag50-PVP and the fact that Ag-NP are subject to transformation, DFM may have overlooked that the smallest Ag50-PVP NP and AMG cannot fully discriminate between different forms of Ag. These limitations need to be considered while discussing the Ag distribution for each single organ in the following paragraphs.

Lymph nodes (LN): It is known that the lymph fluid, and probably also traveling cells within that fluid such as macrophages, enters a lymph node from its periphery via the subcapsular sinus [49]. For small (≤34 nm) and negatively charged fluorescent NP, it has furthermore been shown that they reach mediastinal LN within minutes after instillation into the lung [50]. Although we analysed Ag distribution in mediastinal LN 21 days post instillation, the highest concentrations of Ag were still found in the border regions of many LN. This may reflect the aforementioned sites of entry of lymph fluid, but may also be caused by special macrophages populating the subcapsular sinus [49,51]. It appears also likely that Ag-NP, besides being carried with the lymph fluid, enter the lymph node as alveolar macrophages’ load, as suggested by studies reporting on the progressive transfer of ingested nanoparticles from lung to mediastinal LN [52,53]. In any case, the heavily-stained macrophage-like cells identified by AMG in the subcapsular sinus are fully in accord with all these mechanisms. Interestingly, the high peripheral Ag concentration measured by LA-ICP-MS was not reflected by DFM images, which showed macrophage-like cells with light-scattering material within the medulla of the LN rather than in its periphery (Figure 10i’,j’). It may be speculated that macrophages in the periphery of LN contain high concentrations of Ag+ ions and/or small Ag-NP travelling with the lymph fluid, while macrophages in the depth of the LN may represent those cells that were primarily laden with Ag-NP in the lung. High Ag or Ag^+^ ion concentration of the lymph fluid may also have led to a binding of Ag species to extracellular fibre structures. Such structures span the whole LN and are commonly known as argyrophilic fibres due to their silver ion affinity, as used for routine histology staining protocols. It appears likely that these fibres have adsorbed silver ions from the lymph fluid, making them detectable by AMG and LA-ICP-MS, but not by DFM. Nevertheless, there were small peak-like LA-ICP-MS signals within the centre of LN (Figure 8) reaching an Ag concentration of 200–300 mg/kg, and these small Ag peaks appear to correspond to the Ag-laden phagocytes found by DFM.

Liver: While the majority of local Ag measurements within the liver parenchyma hardly exceeded 50 or 100 µg/kg upon 150 and 300 µg/lung, respectively, Ag hot spots reached 800 or even 20,000 µg/kg. This is equivalent to a 16- and 67-fold concentration of Ag against background, and at least for the 300 µg group, this appears to be an over-proportional increase. By means of DFM of hematoxylin-eosin(HE)-stained sections and also by AMG, we detected light scattering Ag-NP in single cells within the liver parenchyma, which most likely are structural correlates of the high intensity Ag signals noted by LA-ICP-MS. These cells may be Kupffer cells (as mentioned in the Introduction), but further immunocytochemical studies along with high resolution LA-ICP-MS measurements of Ag are necessary. As the number of hot spots was similar in animals treated with 150 or 300 µg Ag50-PVP, it is tempting to speculate that a limited number of anatomically pre-defined binding sites is progressively filled up with particulate matter. This mechanism would be compatible with an involvement of lymphatic vessels, bile ducts and/or Kupffer cells, but certainly needs further confirmation. The focal accumulations of Ag observed by LA-ICP-MS in the liver three and 21 days post administration might correspond to necrotic cells seen in the rat liver 24 h after a high dose of Ag-NP was given intravenously [43]. Of note, the liver has recently been suggested as the organ reacting most sensitively to Ag-NP, a classification based on bile duct hyperplasia [2].

Kidney: The distribution pattern observed in the kidney by LA-ICP-MS was fairly in line with AMG staining as it revealed high Ag concentration located inside the proximal tubuli, a localization that has also been described by Löschner and co-workers [17]. However, in contrast to the latter study, no AMG staining was found in structures associated with the renal filter. Because DFM failed to indicate particular structures in the glomeruli, we believe that Ag50-PVP particles from blood do not accumulate at the renal filter barrier. Instead, we interpret the pronounced occurrence of Ag in the proximal tubuli as a permeation of dissolved Ag ions or Ag-laden albumin. Albumin, which has a diameter of approximately 4.5 nm, is known to permeate the glomerular filter to a low extent and to be removed from the filtrate by a vital re-uptake carried out by proximal tubule cells [54]. Ag-laden proteins may thus accumulate in proximal tubulus epithelial cells and lead to signs of toxicity such as necrosis [45,55], or hyaline degeneration, as observed in rats subjected to the intravenous injection of high doses of Ag-NP [43]. The nature of DFM-positive cells in the kidney cortex, most likely corresponding to the few signals in LA-ICP-MS, is still unknown, but may involve phagocytic cells.

Spleen: Similar to what was found in liver, LA-ICP-MS detected discrete focal accumulations several hundred micrometres in size. Although single DFM-positive cells were noted, their frequency was quite low, such that it is more likely that the focal Ag accumulation measured by LA-ICP-MS corresponds to the round or ovoid structures stained by AMG. Of note, an LA-ICP-MS study on consecutive sections (manuscript in preparation) furthermore showed the three-dimensional extension of similar structures. The size, shape and distribution of the AMG-positive net-like structures resembles follicular dendritic cells. Interestingly, a recent report on altered spleen histopathology upon i.v.-administered Ag-NP showed similarly-sized areas harbouring necrotic lymphocytes along with unusually-stained macrophages [43]. Further characterization work is needed to identify the nature of the cells compromised by accumulated silver.

#### 3.3.3. Estimation of the Silver Burden in Single Cells

Although laser ablation was carried out with a spot size of 50 µm and not at cellular resolution, it appears possible to provide at least a rough estimation of the cellular (or focal) Ag burden using results from calibrated LA-ICP-MS. Thus, under ideal conditions, it appears possible to even extrapolate to a cellular Ag burden, if certain requirements are fulfilled. These would be: (1) the whole amount of silver giving rise to a single signal is concentrated in one cell, i.e., there is no or minimal background staining; (2) the matrices of the standard (chicken liver) and tissue (lymphatic, liver) are identically ablated; (3) the thickness of tissue sections and standards was equal (7 µm); and (4) the portion ablated from a sectioned cell is known, e.g., exactly one half of a spherical cell with a diameter of 14 µm is ablated (50%). A situation likely fulfilling Criteria 1 and 2 was found in liver and also in the medulla of LN, because in these tissues, DFM, AMG and LA-ICP-MS indicated single Ag-NP-containing cells (as shown in Figure 10e,j). Criterion 3 must be assumed as to be fulfilled by the instrument settings during cryo-sectioning, and Criterion 4 can at least be estimated from the size of the sectioned cell. As outlined in the Appendix A, these consideration would lead to an estimation of 7 or 140 pg Ag per cell in a mediastinal lymph node, if the calculation were based on central or marginal peak values, respectively. With respect to quantification, it cannot completely be ruled out that some Ag-NP or Ag^+^ ions were lost from cryo-sections during immersion fixation. However, no major differences were found between the staining patterns of fixed and non-fixed sections of spleen (compare Figure 6i and Figure S3). Nevertheless, compared to our in vitro experiments with cultured alveolar macrophages that were shown to engulf 15–120 pg of several nanomaterials [30], the order of magnitude appears realistic. More detailed studies, especially in the lung, are currently underway.

## 4. Materials and Methods

### 4.1. Particle Characterization

Main particle characteristics and the structure of the PVP coat bound to the surface were previously published [56,57]. PVP was used as a dispersant in the course of Ag-NP preparation [56]. PVP coating is non-toxic and, in contrast to the often used citrate, may bind Ag^+^ ions to a low extent [58,59]. For experiments, silver NP Ag50-PVP and Ag200-PVP NP were diluted from stock solutions, which contained 10% (*w*/*w*) Ag-NP. The primary size and characteristics are shown in Figure S4 and Table S1. Importantly, particles were only mildly negatively charged at pH 7.4 and, therefore, tended to agglomerate under biological conditions, i.e., around pH 7.4.

### 4.2. In Vitro Experiments

The rat alveolar macrophage cell line NR8383 was used for all in vitro experiments. Cells were cultured in 175 cm^2^ culture flasks in F-12K medium (Biochrom GmbH, Berlin, Germany) supplemented with 15% heat inactivated standardized foetal calf serum (FCS) at 37 °C and 5% CO_2_. Experiments were performed with two different protocols: In the first protocol, cells were incubated with NP in serum-free F-12K medium as described [30]. The primary concentration of particles was 180 µg/mL. The suspension was vortexed and ultrasonicated for 10 s with a probe adjusted to 50 W (VibraCell™, Sonics & Materials, Danbury, CT, USA) and further diluted as indicated. In the second protocol, developed by the NanoGEM consortium to primarily avoid high ultrasonic energy input, NP were prepared as suspension (1 mg/mL) and in 10% (*v*/*v*) FCS containing DMEM and stirred at room temperature for 24 h. Stirring was conducted with a magnetic bar at room temperature for 24 h. Cells cultured in F-12K medium were seeded in the 96-well plates and incubated with particles in DMEM containing 10% (*v*/*v*) FCS. Hydrodynamic diameters of Ag50-PVP and Ag200-PVP dispersed with both protocols were measured by optical tracking analysis (Figure S5). Therefore, a NanoSight LM10 instrument equipped with a blue laser (405 nm), an Andor CCD camera and NanoSight tracking Analysis software (NTA 3.1) was used (Malvern Instruments GmbH, Herrenberg, Germany).

All assays were run in 96-well plates (with 3 × 10^5^ cells per well) and repeated three times. Cells were incubated with increasing concentrations of Ag50-PVP or Ag200-PVP for 16 h. Supernatants were harvested, centrifuged (10 min 200 g) and analysed for lactate dehydrogenase activity (LDH) with the Cytotoxicity Detection Kit (Roche Diagnostics GmbH, Mannheim, Germany) according to the manufacturer’s instruction. Untreated cells were used as negative controls. To obtain a positive control, equal numbers of untreated cells were lysed with 0.1% Triton X-100. Optical density was read with a plate photometer (Infinite F200, Tecan Deutschland GmbH, Crailsheim, Germany).

### 4.3. Preparation of Particle Suspension for Instillation Experiments in Rats

Particles were suspended to a final concentration of 1.2 mg/mL 0.9% NaCl and further diluted to required working conditions. Size distribution prior to instillation was determined by optical tracking analyses as described in Section 4.2 (Figure S4).

### 4.4. Animal Experiments and Organ Preparation

Animal experiments were conducted at the animal facility of the University Clinics of Essen, Germany and were ethically approved by LANUV (Dortmund, Germany, Accession Number 84-02.04.2022.A157). Female Wistar rats, strain WU, weighing 200–250 g (Charles River Laboratories, Sulzfeld, Germany), were maintained with a 12-h lights-on lights-off cycle. Food and water were provided ad libitum. For intratracheal instillation, animals (*n* = 5 per group) were briefly anaesthetized with isoflurane. A total volume of 500 µL 0.9% NaCl containing 0 (controls), 0.0375, 0.075, 0.15, 0.30 or 0.6 mg (*w*/*v*) silver NP was intratracheally instilled. In experiments with 0.6 mg particle-free supernatants (volume equivalent to that of the diluted particle suspension) prepared by centrifugation (28,000 *g*, 8 h), they were additionally instilled as control. A Penn Century Microsprayer was used under visual control to apply nebulized suspensions into the trachea. After 3 and 21 days, animals were deeply anaesthetized with a mixture of ketamine and xylazine and bled from the aorta descendens. A cannula was inserted into the trachea, and while the left bronchus was clamped, the right lung lobe was lavaged with 5 × 3 mL 0.9% NaCl to retrieve the broncho-alveolar lavage fluid (BALF). Then, the right bronchus was closed, and the left lung lobe was filled with Cryomatrix (Thermo Shandon Ltd., Runcorn, UK), snap frozen in liquid nitrogen and stored at −80 °C for histologic studies. Liver, kidney and spleen were resected, and representative parts from each organ were weighed in Teflon test tubes to determine their organ Ag content by ICP-MS analysis, which was contracted to Wessling GmbH (Altenberge, Germany). Collections of mediastinal lymph nodes were gathered on small strips of filter paper and covered with cryo-compound before freezing. Liver, kidney and spleen were trimmed, snap frozen in liquid nitrogen and stored at −80 °C until sectioning and subsequent LA-ICP-MS analysis.

### 4.5. BALF Analysis

Cells from pooled rat BALF preparations were collected at the bottom of a centrifuge vial (200 *g*, 4 °C, 10 min). The supernatant was centrifuged again, and the final supernatant was used for protein determination according to the Lowry method using bovine serum albumin as a standard. Fibronectin was detected by a specific ELISA [60]. Final numbers of cells were determined with a Coulter counter (model Z2, Beckman Coulter GmbH, Krefeld, Germany), and the proportion of dead cells was determined by trypan blue testing. Differential cell counting was carried out with cytospin preparations stained with May-Grünewald and Giemsa dyes. At least 400 cells per animal were evaluated under the light microscope.

### 4.6. Immunocytochemistry, Fluorescence Microscopy and Hyperspectral Imaging

Transverse sections (7 µm) were cut from the hilar region of the left lung with a cryo-microtome (Microtome Cryostsat HM 500, MICROM International GmbH, Walldorf, Germany). Sections were dried onto glass slides and stored at −80 °C. To stain for CD68-positive alveolar macrophages, sections were post-fixed with 4% phosphate-buffered formaldehyde for 15 min. Sections were rinsed in phosphate-buffered saline (PBS), and non-specific binding sites were blocked with 3% bovine serum albumin (BSA, fraction V, Serva). An anti-CD68 antibody (AbDSerotec, diluted 1:100 in PBS, 1% BSA) was used and detected with an anti-mouse IgG antibody conjugated to Alexa Fluor^®^488 (Cell Signaling, Danvers, MA, USA, diluted 1:20,000). Sections were rinsed thoroughly in PBS and coverslipped with Roti^®^-Mount FluorCare (Carl Roth, Karlsruhe, Germany), which contained 4′,6-diamidin-2-phenylindol (DAPI) to stain for the DNA of cell nuclei. Micrographs were taken with a Nikon CCD camera (Nikon Lucia 4.0) mounted on an Olympus IX51 fluorescence microscope (Olympus Deutschland GmbH, Hamburg, Germany) equipped with filter sets U-NMU2, UMWiY2 and U-MWIB2 for the fluorescence detection of DAPI, Texas Red and fluorescein isothiocyanate (FITC), respectively.

Phospho-histone γH2AX (Ser139) (20E3) was detected on a 4% formalin-fixed cryo-section (4% PFA/BBS) using a rabbit monoclonal antibody (Cell Signaling Technology, No. #9718), diluted 400-fold in PBS and 1% (*w*/*v*) BSA.

To identify NP in unstained or HE-stained sections, we used an Olympus BX50 microscope equipped with an enhanced dark field condensor and a hyperspectral imaging device (CytoViva Inc., Auburn, AL, USA). Images were analysed with a plugin to ENVI 4.0 Software (CytoViva Inc., Auburn, AL, USA).

For detection of silver in lungs and organs of instilled rats, AMG stainings were carried out according to the detailed protocol by Miller et al. [16]. In brief, this method, originally described by Danscher et al. [61], amplifies metals in tissues in the presence of an electron donator, in this case hydroquinone by reduction of silver ions to metallic silver.

### 4.7. Quantitative Bio-Imaging of ^107^Ag with LA-ICP-MS

#### 4.7.1. Chemicals

Silver nitrate (99.999% pure) was from Sigma-Aldrich Chemie GmbH (Steinheim, Germany). Nitric acid (65%, suprapure^®^) and formaldehyde were from Merck (Darmstadt, Germany). Indium and rhodium standards were from SCP Science (Courtaboeuf, France) or SPETEC VHG Labs (Erding, Germany), respectively. All analytical chemicals used were obtained in the highest quality available. Water was purified through an Aquatron Water Stills purification system Model A4000D (Barloworld Scientific, Nemours, France).

#### 4.7.2. Matrix-Matched Standards and Standard Curve Generation

An aliquot of liver was weighed in a test tube, and after the addition of defined amounts of silver nitrate (AgNO_3_) solution, the mixture was homogenized on ice for 30 s with an Ultra-Turrax (Ika-Werk, Staufen, Germany). The Ag-spiked homogenate was poured into liquid nitrogen resulting in frozen droplets, which were further processed as matrix–matched standard (MMS). Preparation, handling and storing of sections from MMS were essentially the same as for organ sections.

The Ag concentration within these MMS was additionally determined by ICP-MS quantification. Therefore, the MMS were subjected to acidic microwave digestion using a Mars X-press system (CEM Corporation, Matthews, NC, USA) with an optimized protocol (Table S2). Approximately 100 mg MMS were transferred into perfluoroalkoxy (PFA) microwave vessels. Exact weight was determined, and water (2 mL), nitric acid (0.5 mL) and indium solution (0.5 mL, 500 µg/kg) as internal standard were added. Then, the solution was transferred to polymethylene pentene vessels, and rhodium standard solution (0.5 mL, 500 µg/kg) was added to monitor the stability of the ICP-MS system during acquisition. Finally, all samples were diluted with deionized water to a final volume of 25 mL. This procedure was repeated in three independent approaches. The ICP-MS system (iCAP Qc, Thermo Fisher Scientific, Bremen, Germany) was tuned and optimized to achieve maximum signal intensity using a multi-element standard solution. The optimized radio frequency (RF) power was 1550 W with an auxiliary gas flow of 0.8 L/min and a nebulizer gas flow of 1.2 L/min. The system was equipped with a quartz cyclone spray chamber, a PFA µ-flow-ST nebulizer, a quartz injector pipe with an inner diameter of 3.5 mm), a platinum-tipped sampler and a nickel skimmer with an insert of 2.8 mm, respectively. To overcome poly-atomic interference, the kinetic energy discrimination mode was applied adding helium (He) as cell gas containing 6% H_2_ (4.5 mL/min). ^107^Ag and ^109^Ag were detected with dwell times of 50 ms each. Furthermore, ^115^In and ^103^Rh were recorded with dwell times of 10 ms for each element. The Ag concentrations measured this way within MMS are shown in Table 2 (left column).

For LA-ICP-MS analyses, the frozen MMS droplets were embedded in NEG 50™ and cryo-sectioned (7 µm) using a CryoStar NX70 (Thermo Fisher Scientific, Bremen, Germany). Sections were mounted onto glass slides and analysed by LA-ICP-MS (see below). For each standard concentration, 10 lines were ablated corresponding to approximately 1800 data points. The intensity of signals was reflected by the mean count rate per second (cps) and is shown in the right column of Table 1. The resulting standard curve (not shown) was linear within a concentration range of 6.1 × 10^−3^ mg/kg–18.70 mg/kg (*R*^2^ = 0.99991). The limit of detection was 20 µg/kg according to the 3 sigma criterion.

#### 4.7.3. LA-ICP-MS Analysis of Tissue Sections

To image the silver distribution in the embedded tissue sections, tissue sections (7 µm) were post-fixed by immersing the slides into PBS-buffered formaldehyde solution (4%) for 10 min. Slides were then washed 3 times in phosphate buffer and once in deionized water. Micrographs of the fixed sections were obtained with fluorescence or bright field optics of an inverted microscope (BZ-9000, Keyence, Osaka, Japan). A laser ablation system (LSX 213, CETAC Technologies, Omaha, NE, USA) was coupled to an ICP-MS system equipped with a quadrupole mass filter (iCAP Qc, Thermo Fisher Scientific, Bremen, Germany). By means of a pulsed Nd:YAG laser, the samples were ablated at an ultraviolet wavelength of 213 nm. The system was controlled with DigiLaz III software (CETAC Technologies, Omaha, NE, USA). Optimization was performed regarding parameters such as spot size, laser energy, scanning speed, as well as carrier gas flow. Sections were quantitatively ablated in line scans (0 µm gap) using 10% of the full laser pulse energy (3.9 mJ), a scan rate of 50 µm/s, a laser spot size of 50 µm and a pulse repetition rate of 10 Hz. Quantitative ablation was verified microscopically. A carrier gas mix of helium (0.8 L/min) and argon (0.4 L/min) was applied to transfer the ablated dry aerosol to the ICP-MS. To monitor the sensitivity of the system during acquisition, an indium solution (10 ng/L) was continuously introduced into the ICP-MS system by means of a PFA MicroFlow nebulizer and a cyclonic spray chamber using argon as a nebulizer gas. For maximum signal intensity, the ICP-MS instrument was tuned with a multi-element standard solution (10 µg/L: Be, Bi, Ce, Co, In, Li, U). The optimum RF power was 1550 W at an auxiliary gas flow of 0.5 L/min and a sampling depth of 7 mm. A quartz injector pipe with an inner diameter of 3.5 mm and a platinum-tipped sampler and skimmer were used. The isotopes ^107^Ag and ^109^Ag were detected with dwell times of 100 ms each. Image data were further processed and evaluated using ImageJ open source software (website: https://imagej.nih.gov/ij/) and Origin8.0 software (OriginLab Corporation, Northampton, UK).

### 4.8. Statistics

In vitro data were generated in triplicate, and at least three independent repetitions were carried out. Values for each concentration were compared to non-treated controls by two-way analysis of variance (ANOVA) and Bonferroni multiple comparisons test. The half maximal effective concentration (EC_50_) was calculated from nonlinear regression curves calculated by GraphPad Prism 6.01, plotting the logarithmic concentrations of NP concentrations against effects (in % control); the quality of fit was indicated by R^2^ values. For in vivo testing (5 animals per group), all data are expressed as the mean ± standard deviation (SD). BALF data from rats were compared pair-wise to the control group by one-way ANOVA and the post-hoc Dunnett’s multiple comparison test. BALF data from mice were compared by one-way ANOVA followed by the Kruskal–Wallis test. For all experiments, *p* ≤ 0.05 was considered significant.

## 5. Conclusions

Ag nanoparticles (Ag-NP) are cytotoxic to alveolar macrophages in vitro and induce inflammation and, at higher concentration, also genotoxic effects in the lung, as indicated by DNA double-strand breaks. Irrespective of this dose-dependent quality of effects on the lung, Ag-NP in the lung give rise to a distribution of Ag to remote organs, where Ag is detectable in the form of focal accumulations. For the first time, LA-ICP-MS was used to quantitatively image these local accumulations of Ag in peripheral organs, and first estimations emanating from this study suggest that the intracellular dose of Ag is in the picogram range. Unexpectedly, the particulate nature within many silver depots, although mostly found in macrophage-like cells, was not unequivocally confirmed within local lymph nodes, liver, spleen and kidney by DFM. Instead, the combined findings from DFM, AMG staining and LA-ICP-MS drew a more complex image. Thus, nanoparticles with a primary size of far less than 100 nm, agglomerates thereof, but also Ag ion species seem to contribute to focal Ag accumulation. The latter was suggested by massive Ag depots located behind the renal filter barrier. Given the cytotoxic and genotoxic properties of silver ions in high concentrations, focal accumulations in remote organs are a matter of concern, and further studies are needed to unravel the effects up cell populations exposed to high local concentrations of silver species.

## Figures and Tables

**Table 1:** Hydrodynamic diameters of Ag50-PVP and Ag200-PVP dispersed by two protocols.

Particle size (nm)	Ag50-PVP (F12-K, Serum-Free)	Ag50-PVP (DMEM/FCS)	Ag200-PVP (F12-K, Serum-Free)	Ag200-PVP (DMEM/FCS)
Mean ^1^	107.5 ± 0.8	105.6 ± 1.3	175.9 ± 0.7	167.2 ± 0.9
Mode ^1^	96.5 ± 5.0	100.9 ± 2.6	135.4 ± 18.6	154.3 ± 4.9

^1^ Values from three measurements carried out with optical particle tracking.

**Table 2:** Calibration of matrix-matched standards by ICP-MS and LA-ICP-MS.

Ag Concentration Measured by ICP-MS (mg/kg)	Intensity Measured by LA-ICP-MS (Counts per Second)
6.1 × 10^−3^	661
12.5 × 10^−3^	883
93.5 × 10^−3^	1300
25.4 × 10^−2^	2610
42.9 × 10^−2^	2950
89.5 × 10^−2^	5950
18.70	130 × 10^3^

## Supplementary Materials

The following are available online at http://www.mdpi.com/2079-4991/7/12/441/s1: Figure S1: Changes in broncho-alveolar lavage fluid from rat lungs intratracheally instilled with 600 µg of silver nanoparticles; Figure S2: Aspects from BALF and lung tissue after intratracheal instillation of rat lungs with 600 µg Ag50-PVP; Figure S3: Bright field microphotograph of an unfixed spleen cryo-section from a rat intratracheally instilled with 300 µg Ag50-PVP; Figure S4: Size characterization of the silver nanoparticles Ag50-PVP; Figure S5: Hydrodynamic diameter of the silver nanoparticles Ag50-PVP and Ag200-PVP. Table S1: Characterization data of Ag50-PVP and Ag200-PVP; Table S2: Parameters for the microwave digestion of liver samples.

## Appendix A

### Estimation of the Ag Content of a Single Cell under Ideal Conditions

- It is assumed that each signal is retrieved from a tissue volume of 17,500 µm^3^ (50 × 50 × 7 µm^3^); within this volume, there is only one Ag-containing cell sectioned, and no dissolved or particular Ag contributes to the background.
- It is further assumed that the calibration from AgNO_3_ concentration (Ag mass per mass tissue) is valid and the concentrations determined with the matrix-matched standard method for 7 µm-thick sections are correct.
- It is further assumed that the loss of section mass upon drying is of no relevance for the calibration, because these conditions are equally valid also for the sectioned matrix-matched standard material.
- Then, a typical Ag signal measured in the lymph node’s medulla (see Figure 8c) is = 200 µg/g.
- Assuming a density of 1 g/mL, the mass of the ablated area per laser shot (before drying) is: 50 µm × 50 µm × 7 µm/thickness = 17,500 µm^3^ = 17.5 ng.
- For the measured peak value of 200 µg Ag/g, the Ag mass contained in the ablated mass is calculated as: 200 µg/1,000,000 µg = *X*/0.0175 µg. Thus, *X* = 0.0000035 µg = 3.5 pg.
- Given that a cell has a thickness of 14 µm (typical for, e.g., a macrophage), the above value doubles to 7 pg for a single sectioned DFM-positive cell.
- According to this calculation, the peak value found in an LN (4000 µg/g (see Figure 8c) would correspond to a cell burden of 140 pg.
