# Supplementary material for: Silver Nanoparticles in the Lung: Toxic Effects and Focal Accumulation of Silver in Remote Organs
Source: Nanomaterials (Basel). 2017 Dec 12;7(12):441. doi: 10.3390/nano7120441 (PMC5746931; doi:10.3390/nano7120441)
Supplement: Supplementary file 1 [file nanomaterials-07-00441-s001.pdf]

nanomaterials

Silver Nanoparticles in the Lung: Toxic Effects and Focal Accumulation of Silver in Remote Organs

Supplementary Material

Martin Wiemann [1]*, Antje Vennemann [1], Franziska Blaske [2], Michael Sperling [2], Uwe Karst [2]

1 IBE R&D Institute for Lung Health gGmbH, Mendelstr. 11, 48149 Münster, Germany; vennemann@ibe-ms.de
2 Institute of Inorganic and Analytical Chemistry, University of Münster, Corrensstr. 30, 48149 Münster, Germany; franziska.blaske@googlemail.com; msperlin@uni-muenster.de; uk@uni-muenster.de
* Correspondence: martin.wiemann@ibe-ms.de; Tel.: +49-251-9802340

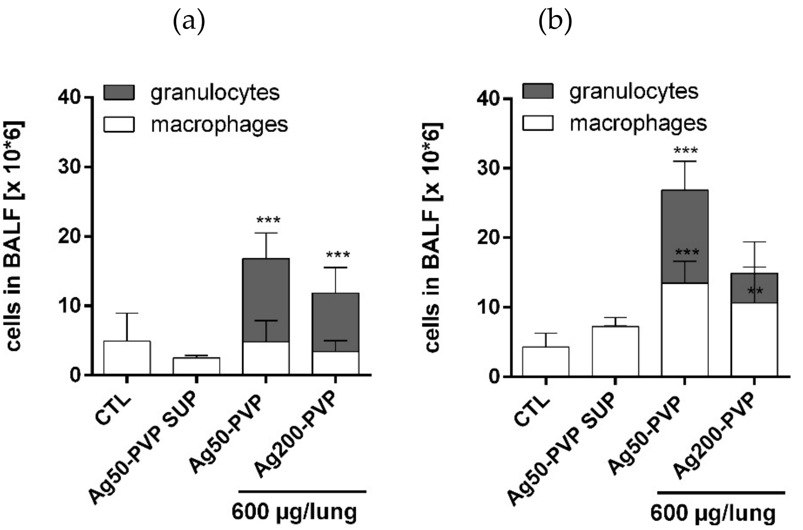

Figure S1. Changes in broncho-alveolar lavage fluid from rat lungs intratracheally instilled with 600 µg of silver nanoparticles. Comparison of Ag50-PVP and larger Ag200-PVP with the particle diminished supernatant (Ag50-PVP SUP) prepared from Ag50-PVP and vehicle treated control are shown after 3 (a) and 21 days (b). Note that at both time points the significant increase of granulocytes and macrophages versus vehicle-treated animals (CTL) was larger for Ag50-PVP. No effects were noticed for the particle diminished supernatant Ag50-PVP SUP vs. vehicle-treated animals; **= $p \leq 0.01$, ***= $p \leq 0.001$, according to one way ANOVA.

.

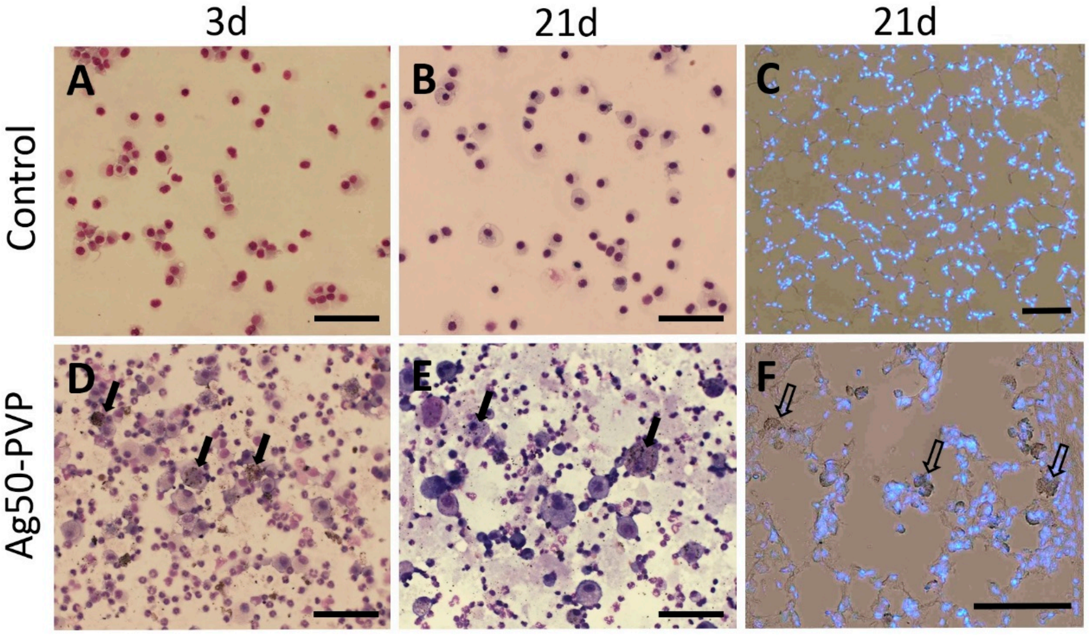

Figure S2. Aspects from BALF and lung tissue after intratracheal instillation of rat lungs with 600 µg Ag50-PVP. Pappenheim-stained cytospin preparation of BALF cells from vehicle-treated (A, B) and Ag50-PVP-treated rats (D, E) after 3 and 21 days. (C, F) show a vehicle- (C) and Ag50-PVP-treated lung (F) stained for nuclear DNA with DAPI (blue), a low bright field illumination was added to show lung structure. Ag50-PVP increases inflammatory cells in BALF and locally destroys septal structure of the lung; black arrows in (D, E) point to Ag-laden macrophages; open arrows in (F) indicate Ag-laden macrophage-like structures bordering compromised lung areas.

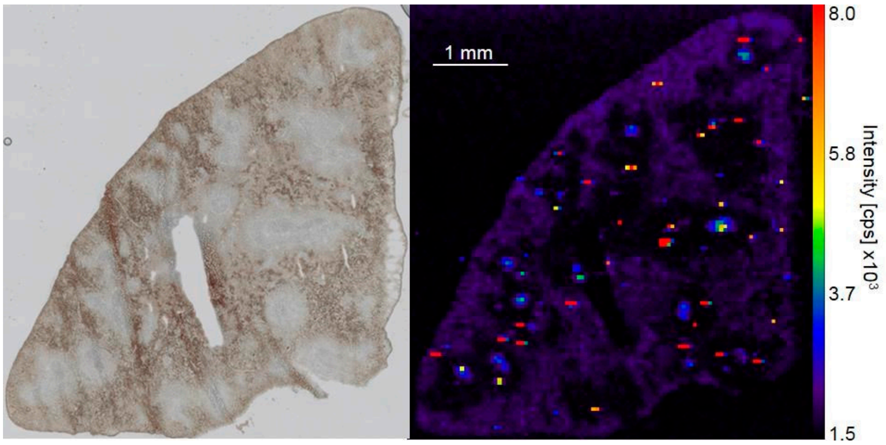

Figure S3. Bright field microphotograph of an unfixed spleen cryo-section from a rat intratracheally instilled with 300 µg Ag50-PVP. Left: Red and white pulp can be easily distinguished. Right: Non-calibrated silver distribution map of the respective spleen section; note the high congruency of the low silver signal (^{107}Ag) with the red pulp; intense spots are mainly found in the white pulp**.**

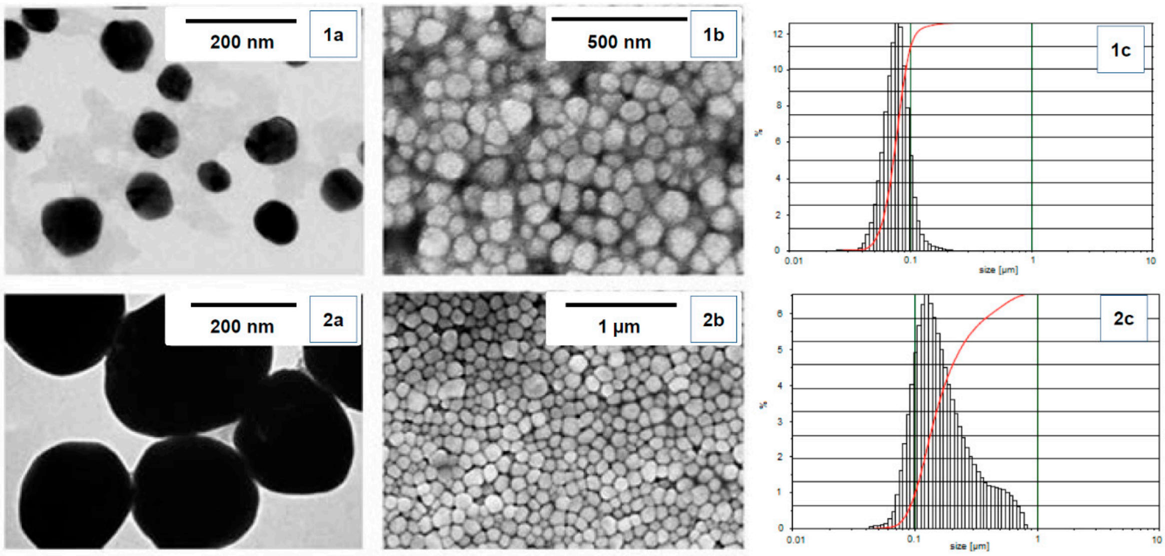

Figure S4. Size characterization of the silver nanoparticles Ag50-PVP. Upper and lower images show data for Ag50-PVP (1) and Ag200-PVP (2), respectively. (1a, 2a) transmission electron microscopy, (1b, 2b) scanning electron microscopy, (1c, 2c) analytical ultracentrifugation (Images from [53], with permission).

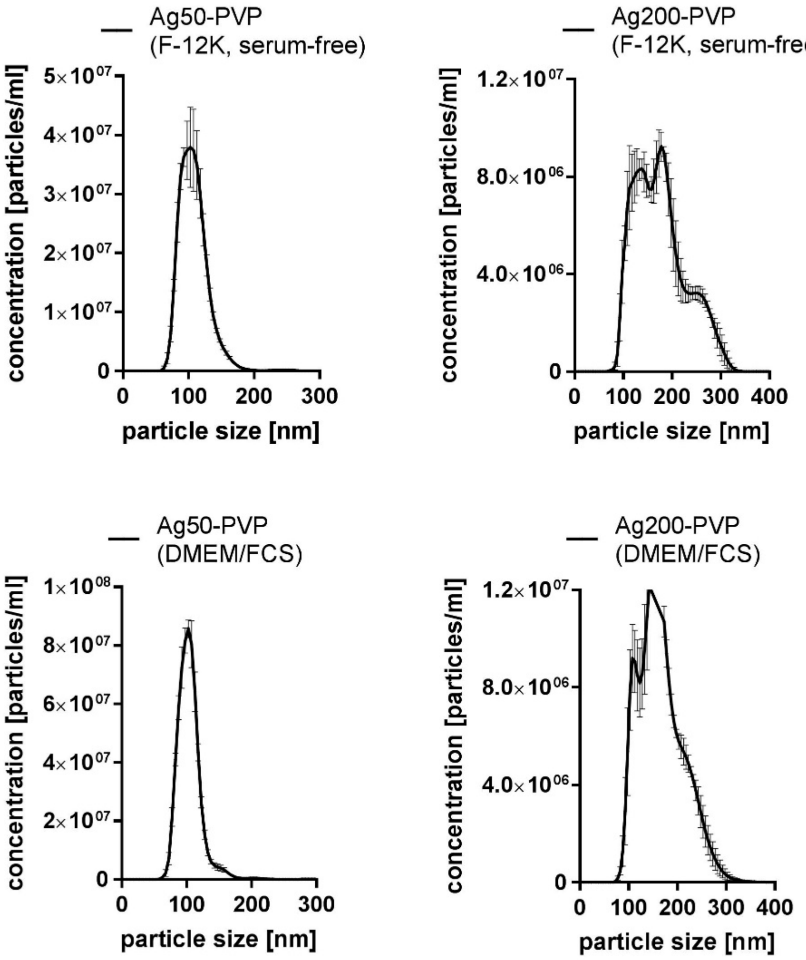

Figure S5. Hydrodynamic diameter of the silver nanoparticles Ag50-PVP and Ag200-PVP. Particles were dispersed in either serum-free F-12K medium (upper graphs), or in DMEM containing 10% fetal calf serum (FCS) using the stirring protocol (lower graphs). All particle suspensions were diluted at least 100-fold with serum-free medium (F-12K or DMEM) to avoid interference of serum proteins with optical particle tracking. Curves were averaged from three measurements whose mean and mode values are shown in the main text.

Table S1. Characterization data of Ag50-PVP and Ag200-PVP [53].

	**Ag50-PVP**	**Ag200-PVP**
**Shape**	Quasi spherical	Quasi spherical
**Concentration**	10 % (wt/wt)	10 % (wt/wt)
**Size/size distribution & aggregation/ agglomeration state**	DLS-agglomerates size: 123 nm<br>TEM: d_{50}= 79 nm, d_{90}= 99 nm<br>AC: d_{50}= 77 nm, d_{90}= 101 nm	DLS-agglomerates size: 408 nm<br>TEM: d_{50}= 134 nm, d_{90}= 300 nm<br>AC: d_{50}= 95 nm, d_{90}= 188 nm
**Crystal structure**	Cubic	Cubic
**Surface chemistry**	XPS: Atom% C 59.1, O 17.5, Ag 15.9, Na 7.5<br>SIMS: Ag, Cl, $C_xH_yO_z$	XPS: Atom % C 77.2, O 10.7, Ag 0.4, N 11.7, Na 1.0<br>SIMS: $C_xH_yO_z$
**Surface charge**	Zeta potential: - 12.5 mV+/- 0.5	Zeta potential: - 5.5 mV+/- 0.5

TEM: transmission electron microscopy; BET: nitrogen adsorbing surface according to the Brunauer-Emmett-Teller equation; AC: analytical ultracentrifugation; XPS: X-ray photoelectron spectroscopy (Table taken from [53], with permission)

Table S2. Applied parameters for the microwave digestion of liver samples

Parameter	#1	#2	#3	#4	#5	#6	#7
Time [min]	5	10	15	20	30	35	40
Temperature [°C]	20	100	100	150	150	175	175
Power [W]	-	800	800	800	800	800	800
